# Low-Temperature Thermal Degradation of Disinfected COVID-19 Non-Woven Polypropylene—Based Isolation Gown Wastes into Carbonaceous Char

**DOI:** 10.3390/polym13223980

**Published:** 2021-11-17

**Authors:** M. M. Harussani, Umer Rashid, S. M. Sapuan, Khalina Abdan

**Affiliations:** 1 Advanced Engineering Materials and Composites Research Centre (AEMC), Department of Mechanical and Manufacturing Engineering, Universiti Putra Malaysia, Serdang 43400, Selangor, Malaysia; mmharussani17@gmail.com; 2 Institute of Nanoscience and Nanotechnology (ION2), Universiti Putra Malaysia, Serdang 43400, Selangor, Malaysia; 3 Laboratory of Biocomposite Technology, Institute of Tropical Forestry and Forest Products, Universiti Putra Malaysia, Serdang 43400, Selangor, Malaysia; khalina@upm.edu.my

**Keywords:** slow pyrolysis, COVID-19 isolation gown, polypropylene, char, pyrolysis parameters

## Abstract

Yields of carbonaceous char with a high surface area were enhanced by decreasing the temperature to improve the conversion of hazardous plastic polypropylene (PP), the major component in abundantly used isolation gowns. This study applied pyrolysis with different low pyrolytic temperatures to convert disinfected PP-based isolation gown waste (PP-IG) into an optimised amount of char yields. A batch reactor with a horizontal furnace was used to mediate the thermal decomposition of PP-IG. Enhanced surface area and porosity value of PP-IG derived char were obtained via an optimised slow pyrolysis approach. The results showed that the amount of yielded char was inversely proportional to the temperature. This process relied heavily on the process parameters, especially pyrolytic temperature. Additionally, as the heating rate decreased, as well as longer isothermal residence time, the char yields were increased. Optimised temperature for maximum char yields was recorded. The enhanced SBET values for the char and its pore volume were collected, ~24 m^2^ g^−1^ and ~0.08 cm^3^ g^−1^, respectively. The char obtained at higher temperatures display higher volatilisation and carbonisation. These findings are beneficial for the utilisation of this pyrolysis model in plastic waste management and conversion of PP-IG waste into char for further activated carbon and fuel briquettes applications, with the enhanced char yields, amidst the COVID-19 pandemic.

## 1. Introduction

Severe acute respiratory syndrome coronavirus (SARS-CoV-2 or COVID-19 virus) pandemic attacked the world vigorously from the fourth quarter of 2019 until the present. Thus, the World Health Organization (WHO) [1] announced a public health emergency due to the outbreak on 30 January 2020. As reported on 7 February 2021, there were 106 million active COVID-19 cases, with 2.3 million deaths calculated from 219 countries and regions affected by the global outbreak [2]. Environmental pollution is one of the most worrying consequences due to this COVID-19 epidemic. As of 22 November 2020, a massive amount of COVID-19 medical waste (CMW), approximately 54,000 tons of waste, had been produced daily [3]. In this context, the CMW is defined as medical waste that has been used in treating COVID-19 patients in the hospitals and healthcare facilities, which comprises personal protective equipment (PPE), such as face masks, non-woven polypropylene-based isolation gowns, gloves, goggles, and disinfectant containers. Specifically, the amount of medical waste generated during the COVID-19 crisis has increased dramatically to almost four times the usual situation in Hubei province. Furthermore, the CMW in Wuhan was recorded at a peak of roughly 240 t/day and indeed, the incineration capacity is around five times greater [3]. Thus, this scenario leads to increasing global production of plastics waste.

Plastics are synthetic organic materials made up of polymers, consisting of long molecules built around chains of carbon atoms, generally hydrogen, oxygen, sulphur, and nitrogen filling. Production of plastic wastes increases every year, with 381 million tonnes as recorded in 2020 [4,5], which includes various types of plastics such as high-density polyethylene (HDPE), low-density polyethylene (LDPE), polyethylene terephthalate (PET), polyvinyl chloride (PVC), polystyrene (PS), and polypropylene (PP) [5,6]. It is noted that PPE and N95 masks utilised by HCWs and publics include plastics as major constitutes, representing 25% by weight [7]; whereas widely used facemasks and other PPEs, including protective suits, isolation gowns and gloves, are mainly made up of PP (about 72%) [8]. According to the Ministry of Health Malaysia (MOH) [9], PPEs, e.g., isolation gowns and face masks that have been used widely by healthcare workers in Malaysia, are made from non-woven fabric PP polymer. In current situations, the practice used to decompose them is via burning and incineration methods, where various significant atmospheric pollutants were released. This is due to these plastics containing additives such as colorants, plasticisers, and stabilisers. These cadmium- and lead-containing materials are hazardous to the environment. Therefore, proper green technology is paramount in transforming these hazardous wastes into wealth; as the abnormal situations happened, the urgent requirement of the existing treatment system to cope with the significant changes must be prioritised.

Pyrolysis is a thermal degradation approach of plastic materials conversion into three major product forms, includes gaseous, liquid (oil), and solid (char), at elevated temperature under a deoxygenated environment [10]. The products mentioned above are valuable for industries, especially green production and refineries. Generally, gas produced from the pyrolysis has significant calorific value as well as high potential to be utilised as a heating source in industrial pyrolysis plants, converted as chemical feedstock for polyolefins production, and used in combustion (gas) turbines to generate electricity [11]; whereas liquid oil yielded via fast pyrolysis with a higher temperature has wide and multiple applications, including furnaces, boilers, diesel engines, and turbines [12]. High-quality solid char formation in the slow pyrolysis approach is higher than in fast pyrolysis. Furthermore, its higher calorific value and low sulphur content has made it ideal for combustion with coal or other natural binders [13,14], used as fuel, filler in composite and building materials [15,16], and water adsorbent in water treatment [17]. Temperatures, type of reactors, time of sample residence, pressure, heating rate, process flow rate, type of fluidising gas, and catalysts are the main parameters affecting the end product of the pyrolysis. From previous studies, pyrolysis of plastic is generally carried out at final temperature for about 500 °C (moderate temperature) with lower heating rate (~10 °C/min) using a batch reactor [14,18,19,20,21,22,23,24], with these requirements leading to higher char yields; whereas fast pyrolysis contributes to higher gaseous and fuel-like liquid yields [13,25,26,27]. Recently, there have been several review articles [28,29,30] that summarised the proper plastic wastes treatment in detail.

Literature reviews on the application of slow pyrolysis in PP plastic conversion are few to the best of our knowledge [23,24,31,32,33,34,35]. Demirbas et al. [36] investigated the relationship between the yield distributions of the pyrolytic products collected from PP with the different pyrolysis temperatures. The authors observed that the increasing pyrolysis temperature led to lower total olefin fraction yields, while contributing to higher aromatics fraction yields. The pyrolysis experiment of the 3 mm-size PP powder was carried out in a batch reactor with a heating rate of 10 °C/min in deoxygenated conditions. At pyrolysis temperature of 740 °C of PP waste, the gas, oil, and solid residue collected were 49.6, 48.8, and 1.6 wt.%, respectively. From the results obtained, the authors had suggested practicing pyrolysis via lower temperature and reduced heating rate to optimise the solid residue yields. This statement is supported by the works of Ahmad et al. [33], as they studied the product yields as a function of pyrolysis temperature. The total solid residue of 1.34 wt.% was achieved at 300 °C with a higher heating rate of 20 °C/min. Sogancioglu et al. [37] slow pyrolysed pp plastic waste to yield chars for filler applications using a batch reactor. Approximately 2.67% of chars were obtained from low pyrolysis temperature and slow heating rate. Wong and Broadbelt [38] investigated the influence of residence time by fixed two pyrolysis temperatures of 350 °C and 420 °C of PP and PE plastics for 2 h and 18 h, respectively. The authors found that longer residence time increased the consistency of the product with lighter and lower molecular products, which is suitable for fuel and chemical applications.

To optimise the pyrolysis parameters to maximise the char residue yields, an insight into the applicability of a detailed analysis considering temperature and reaction time may be useful. Char residue yielded from pyrolysis of disinfected PP-based isolation gown waste (PP-IG) has its own significant structural, mechanical, and surface area properties. In this study, a comprehensive analysis of the effect of temperature on the char yields and specific surface area values via the facile pyrolysis process to degrade PP plastic waste will be presented. As for novelties, these findings will contribute to understanding of the applicability and impediments of these hazardous waste materials generated from hospitals, PP-IG derived char as solid precursors for activated carbon/graphene production, and also fuel briquette applications while promoting environmentally friendly and more efficient waste-to-wealth conversion ways.

## 2. Materials and Methods

### 2.1. Polypropylene (PP) Isolation Gown Waste

The pyrolysis experiments were performed with 0.25 mm PP-IG samples. The PP-IG samples were prepared from used PPEs, including isolation gowns. Next, the collected materials were shredded using FRITSCH Universal Cutting Mill PULVERISETTE 19, Idar-Oberstein, Germany, with 0.25 mm perforation sieve cassette into 0.25 mm-size PP-IG samples. The samples then were directly used for sample characterisations and experiments of laboratory-scale pyrolysis with specified parameters.

For a comprehensive overview, the PPEs, include the isolation gowns, hairnets, shoe covers, and face masks, were collected from Universiti Putra Malaysia (UPM) Healthcare Centre (PKU), UPM Serdang, Selangor, Malaysia. Priors to shredding steps, the collected waste PPEs were disinfected thoroughly under the supervision of HCWs from UPM PKU staff. Materials collected were cleaned and disinfected using normal soap meticulously. The materials were dried under direct sunlight and shredded using the abovementioned cutting mill into small powder of size ~0.25 mm for better pyrolysis experiment. Twenty g of segregated PP-IG samples were filled into a quartz boat and placed at the centre of the furnace. The elemental composition of PP plastic is as shown in Table 1.

### 2.2. Preparation of Char

A slow pyrolysis study was executed for PP-IG samples using a tube furnace batch reactor (Carbolite) at a fixed heating rate of 3 °C/min with 1.5 atm pressure under nitrogen gas environment (99.9992% purified N_2_ gas). Prior to the thermal degradation study, thermal-gravimetric analysis (TGA) and derivative thermogravimetry (DTG) analysis were performed in order to determine the optimum temperature in which degradation of PP-IG samples occurs.

**Figure 1:**
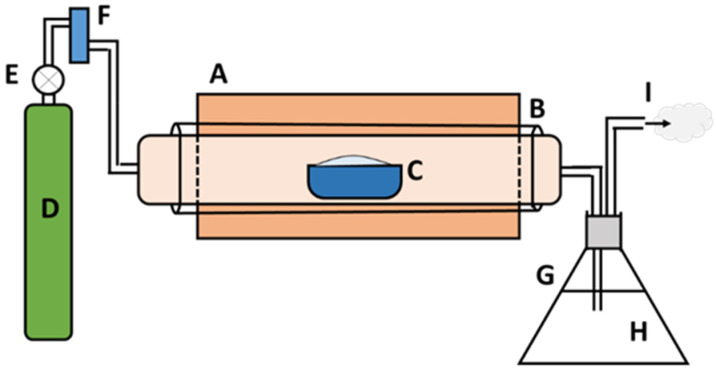
The schematic illustration of pyrolysis system setup: (**A**) electrical furnace, (**B**) ceramic tube, (**C**) alumina boat, (**D**) N_2_ gas cylinder, (**E**) gas regulator valve, (**F**) flow rate meter, (**G**) conical flask, (**H**) water, and (**I**) outlet gas.

Based on the TGA and DSC data, five pyrolysis temperatures (450, 500, 550, 600, and 650 °C) were used as the degradation range of PP-IG samples was from 400–550 °C. For a better understanding, the following processing procedure was applied: 20 g PP-IG samples were filled into alumina boats for each run and then inserted into the ceramic tube furnace. N_2_ gas with a 1.5 cc/min flow rate was purged continuously in order to produce an inert environment inside the isolated reactor. Thus, it also helped to expunge the unwanted pyrolysis vapour products. The gas outlet pipe was then passed through a conical flask filled with water, which was used to detect any leakage throughout the system. The schematic diagram of the experimental setup is represented in Figure 1. A fixed lower heating rate with a residence time of 5 h was used to produce the maximum amount of char yields. Next, the amount of char (by weight) was collected and measured using an electronic balance. The char yields (wt.%) were calculated with Equation (1) and recorded, which was then analysed.

(1) $$Solid\;product\;yield=\frac{m_{product}}{m_{waste}}\times 100\%\;$$

where $m_{product}$ is the amount of char and $m_{waste}$ is the pyrolysed waste amount.

### 2.3. Characterisation of Char

Qualitative and quantitative characterisation of char was carried out. Morphological and structural analyses were carried out using analytical techniques for the yielded char like field emission scanning electron microscopy (FESEM, FEI, Sydney, NSW, Australia) and energy dispersive X-ray (EDX, Oxford Instruments, Oxford, UK); whereas the compositional analysis was conducted via the analytical approach of Fourier transform infrared spectroscopy (FTIR, Thermo Fisher Scientific, Waltham, MA, USA) and Raman spectroscopy. Next, the physical properties of specific surface area were measured using Brunauer–Emmett–Teller (BET, Micromeritics Instrument, Norcross, GA, USA) analysis, while its thermal properties were analysed via thermal-gravimetric analysis (TGA, Mettler Toledo, Shah Alam, Selangor, Malaysia) and derivative thermogravimetry (DTG, Mettler Toledo, Shah Alam, Selangor, Malaysia).

#### 2.3.1. Proximate Analysis

The proximate analysis was performed using TGA to obtain the moisture, ash, volatile matter, and fixed carbon contents; whereas Equation (2) was used for calculating the fixed carbon. The moisture content has been overlooked since the result of TGA depends on a dry basis [39].

(2) Fixed carbon (%)=100−Volatile Matter−Ash Content 

#### 2.3.2. FESEM

Nova NanoSEM 230 FESEM (FEI, Sydney, NSW, Australia) is used to view the specified area of the samples via 1 nm resolution at 15 kV. Secondary electron (SE) imaging can be undertaken in field-free and immersion mode (TLD) for comprehensive low-to-high resolution imaging (100,000× magnification). The FESEM service has been accredited by the Quality System of MS ISO/IEC 17,025 Certification.

#### 2.3.3. EDX

Max 20 EDX (Oxford Instruments, Oxford, UK) was used to determine the elemental composition of specific points or to map out the lateral distribution of elements from the imaged area. EDX has combined with FESEM above in order to provide the elemental analysis on a specified area.

#### 2.3.4. FTIR

The FTIR analysis adopted an attenuated total reflective (ATR) technique with a wavelength range of 400 to 4000 cm^−1^ to identify functional groups in the char materials. The solid chars collected from various pyrolytic temperatures were characterised by using a Nicolet 6700 FTIR Spectrometer (Thermo Fisher Scientific, Waltham, MA, USA). In addition, the quality or consistency of the samples could be determined.

#### 2.3.5. Raman Spectroscopy

WITec Alpha 300R Raman Spectroscopy (Ulm, Germany) was used to determine the chemical structure of the samples and to identify the compounds present by measuring their molecular vibrations. A 488 nm laser excitation with a single spectrum type of analysis was used to characterise the char samples.

#### 2.3.6. BET

Micromeritics’ Tristar II Plus BET equipment (Micromeritics Instrument, Norcross, GA, USA) was used to carry out BET surface area analysis, which provides the value of the specific surface area, pore volume, and pore size distribution of solid char via nitrogen adsorption measured as a function of relative pressure. For a comprehensive understanding, a calculation of the volume of adsorbed gas corresponding to a monomolecular layer on the surface of the samples shall decide the surface area. This technique, therefore, covers essentially external area and pores to assess the total surface area. Thus, the BET surface area was used to determine the material porosity, from solid microporous materials to mesoporous materials.

#### 2.3.7. TGA-DTG

Thermal degradation was employed to measure the changes in characteristics of the char with increased temperatures using Mettler Toledo’s TGA-DSC HT 3 equipment (Mettler Toledo, Shah Alam, Selangor, Malaysia). TGA was performed at a range of temperatures, 25 to 600 °C, with a heating rate of 10 °C/min.

### 2.4. Statistical Analysis

SPSS software, a statistical tool, was used to do a variance analysis (ANOVA) on the weight percentage of yielded chars. Duncan’s test was used for mean comparison at a significance level of 0.05 (*p* ≤ 0.05).

## 3. Results and Discussions

### 3.1. Proximate Analysis of PP-IG Samples as Feedstock

Table 2 shows the proximate analysis of the raw PP-G samples used in the experiment. The PP-IG samples were characterised by a low ash content (0.39%) as well as a high volatile weight percentage. The study showed that the raw PP-IG samples needed further processing before energy generation was applied due to their high volatile material. According to Hersztek et al. [40], high volatile matter content significantly affects the combustion process, and since the composition is higher than coal, specific methods for combustion of the plastic wastes are needed.

### 3.2. Effect of Pyrolysis Temperature towards Char Yields and Its Thermochemical Performance

**Figure 2:**
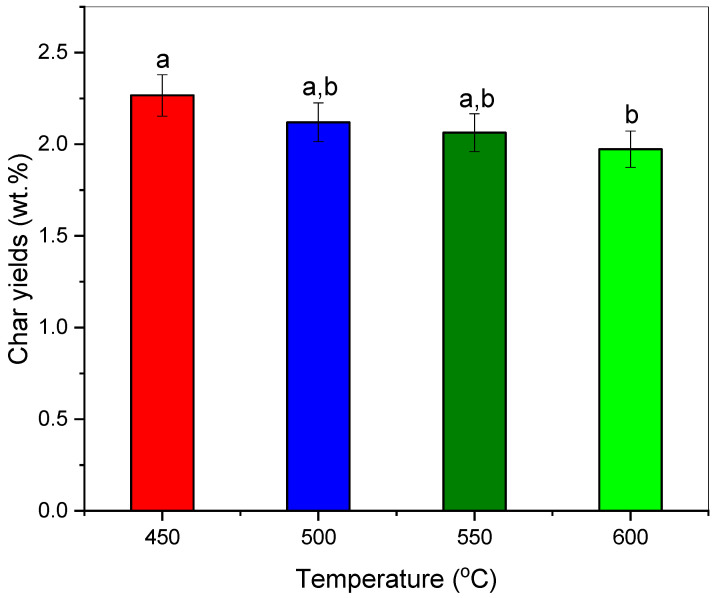
Char yields (wt.%) at different pyrolytic temperatures (°C). Take note that ^a,b^ values with different letters in the same column are significantly different (*p* < 0.05).

The amounts of char obtained through the experiments in lab pyrolysis from PP-IG powder are described in Table 3 and Figure 2. At a lower pyrolytic temperature, char yield was significantly elevated and the highest. The pyrolysis of PP-IG samples at 450 °C produced the highest amount of carbon char of 2.27 wt.%, compared to other different process temperatures. According to Witkowski et al. [41], this is due to a slower and inefficient decomposition of propylene trimer at a lower temperature than that of a higher temperature, where the decomposition, including the weakening of polymer PP chain branches and its backbone double bonds, begin at 387 °C. Therefore, low temperature (450 °C) resulted in a greater amount of char, but the heating value of the char increased with temperature [42]. This parameter needs to be taken into account for fuel applications and soil amendment agents for agriculture. This result was twice the char yields by discoveries from Ahmad et al. [33] and Demirbas et al. [36] with 1.34 and 1.6 wt.%, respectively. Thus, it shows that the application of low process temperature and heating rate of pyrolysis, as well as longer residence time via batch reactor, are significant in improving the yield of solid char. Other than char residue, there is also waxy residue at the mouth of the ceramic tube, which could be utilised as alternative lubricant base-stock and further refining such as dewaxing for industrial applications.

### 3.3. Characterisation of Solid Product Char and PP-IG Samples

#### 3.3.1. Volatile Matter Content

Table 4 displays the contents of moisture and volatile matter of char samples at various pyrolytic temperatures. There was no significant moisture content difference across all samples from the observations, where the moisture content varied only at the range of 0.39 to 0.67 wt.%. In addition, the volatile matter was significantly low for all chars, with 450 °C char having 9.47 wt.% of volatile matter. With the rising of pyrolysis temperature up to 600 °C, the volatile matter content seemed to be declining [33,36].

#### 3.3.2. Surface Morphology Analysis

**Figure 3:**
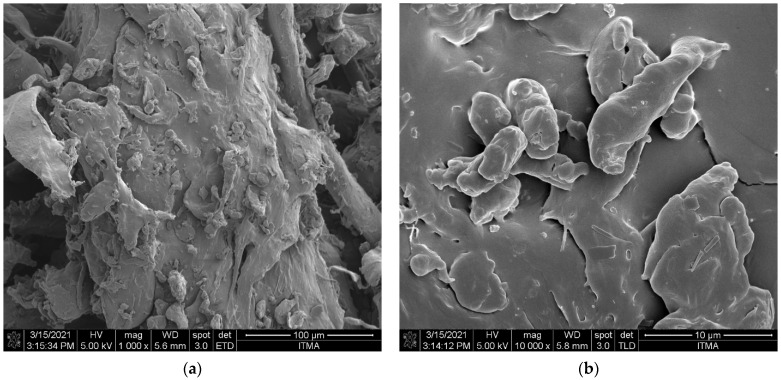
FESEM images of PP-IG samples at magnifications of (**a**) 1 kx and (**b**) 10 kx.

**Figure 4:**
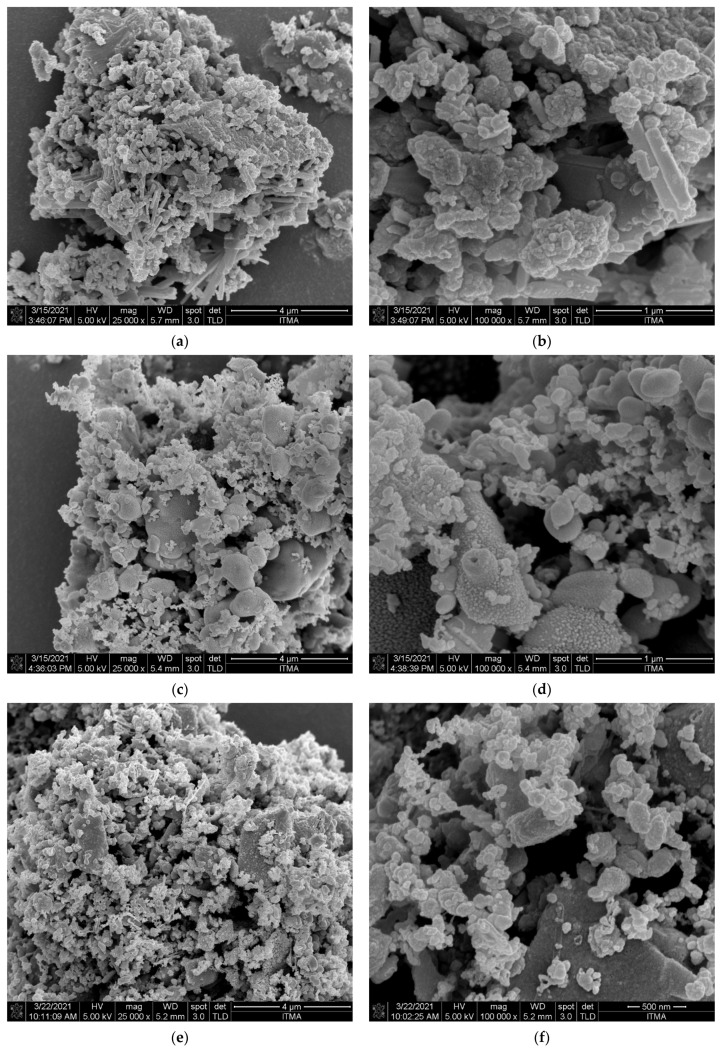

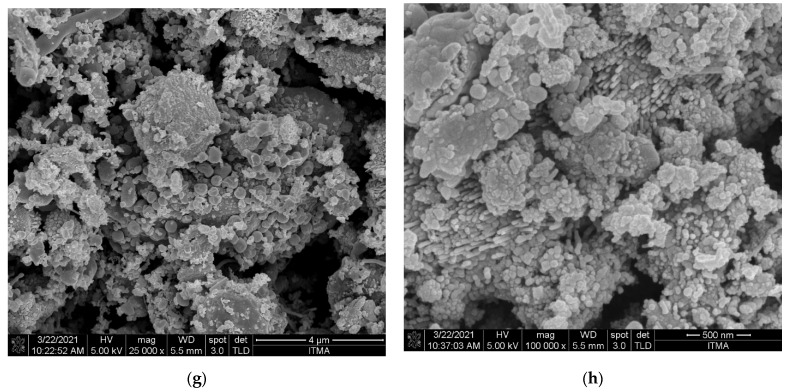
FESEM images of C-450 at magnifications of (**a**) 25 kx and (**b**) 100 kx; C-500 at magnifications of (**c**) 25 kx and (**d**) 100 kx; C-550 at magnifications of (**e**) 25 kx and (**f**) 100 kx; C-600 at magnifications of (**g**) 25 kx and (**h**) 100 kx.

To investigate how the thermal decomposition and the pyrolysis temperature affected the morphology of the PP-IG samples and chars, FESEM analysis was carried out for the materials. The FESEM images shows the raw polypropylene and chars pyrolysed at various temperatures. From our observations, the char particles revealed different size and shapes which associated to the pyrolysis temperature and sample preparation [43]. The raw sample of PP waste indicated a rough and non-porous structure with fairly homogenous polymer [44], as shown in Figure 3. In comparison, Figure 4 presents FESEM micrographs of char samples of C-450, C-500, C-550, and C-600, with various pyrolysis temperatures.

The structure of most char samples is spherical. The observations from FESEM images were in agreement with Sharma et al. [45] and Sogancioglu et al. [37]. The depolymerisation of waste PP increased with the temperature rise. C-600 demonstrated smaller spherical particle size distribution compared to the others. On the other hand, the surface of char samples revealed irregularity along with the increasing pyrolysis temperature, which was associated with depolymerisation. C-600 showed the best depolymerisation compared to C-450, C-500, and C-550. From Figure 4a, the average particle size distribution of char samples was between 0.2 ad 0.8 μm. However, there were some larger size particles within the samples. Moreover, higher magnification micrographs (Figure 4b) displayed clearer images of porous structures within the char samples (C-500, C-550, and C-600). Due to organic material volatilisation, the presence of deep channels and pores became more noticeable with an increased temperature [19]. However, there was no destruction of the porous structure observed at a higher temperature which was related to increased carbonisation reaction. According to Sogancioglu et al. [37], the pyrolytic carbon deposit that occured from carbonisation reactions might cause the other particles to exist. The hydrocarbons were released as volatile substances from the chars during those reactions, and Table 3 provides a description of the volatile matter in the samples. The interaction of these volatile matters with pores could lead to the deposition of crack and carbon [46,47].

For instance, the formation of pores is a key factor that regulates the final use of char for fuel or gasification, as the contribution of micropores is increased by the increase in the pore enlargement that affects the specific surface area of chars. The increase of the efficient surface area helps to increase the reactivity of chars during the transformation phase.

The elements’ content of both raw PP-IG samples and chars generated from EDX results is displayed in Table 5. In both raw and carbon samples, the dominant element is carbon, accompanied by oxygen and other low-weight percentage elements. The raw PP-IG to char samples showed decreases in carbon content which contributed by increasing pyrolysis temperature from 450 °C to 600 °C. There was a slight increase in carbon content from C-500 to C-550. On the other hand, PP-IG samples have a low percentage of oxygen content, 4 wt.% compared to the char samples. For chars, the oxygen content increased with increased process temperature. Thus, it led to the increase in the high heating value (HHV) of the char yielded, based on Selvarajoo and Oochit [39]. Conclusively, increased pyrolysis thermal temperatures resulted in decreased char carbon density, explaining the decreasing percentage of the weight of char yields and encouraging oxygen adsorbed by decreased dehydration and volatility rates. Table 5 also lists other elements. Basu [48] reported that P, Al, Ca, and K are the primary materials of ash that formed during the pyrolysis as parts of the char yielded.

#### 3.3.3. Surface Area and Porosity Measurement

Textural properties of char and neat PP-IG samples were calculated at 77 K via the processes of adsorption and desorption of N_2_. Table 6 represents the specific surface area and characteristics of porous structure in the char samples at various elevated temperatures. As the pyrolysis temperature rose, a porous structure in the chars was formed due to the devolatilisation of PP plastic waste. In addition, the carbonisation of the polymer at elevated temperature also contributed to the increase in carbon content as well as improving the material porosity in the chars [49,50]. From our observations, when the temperature was increased from 450 to 600 °C, the pore size increased as the volatile matter were shattered, and the pores indicated the void spaces in chars [37]. Generally, chars yielded at the temperature of 600 °C (C-600) demonstrated a higher surface area (S_BET_), pore size (D_p_), as well as pore volumes (V_p_) compared to C-450, C-500, and C-550. The surface area, pore volumes, and pore sizes of char were improved by approximately 28%, 30%, and 70%, respectively, from 450 °C to 600 °C. Moreover, several previous literature [13,37] had represented a similar trend of improvements as the temperatures were raised. However, the S_BET_ quality of yielded waste-derived chars, in our works, was enhanced by more than 38% and 40% compared to PP char collected by previous works [13,37].

#### 3.3.4. Thermal Gravimetric Analysis (TGA)

**Figure 5:**
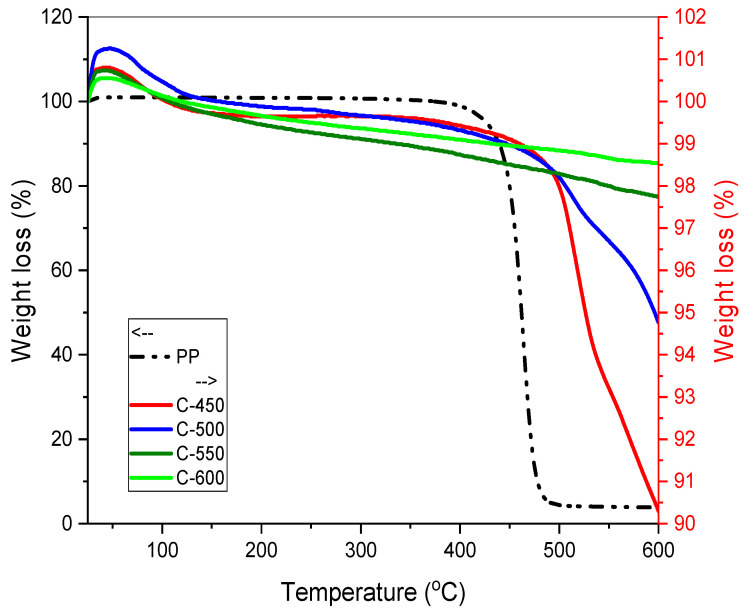
TG curves for raw PP-IG samples, C-450, C-500, C-550 and C-600 with a temperature range of 25–600 °C.

**Figure 6:**
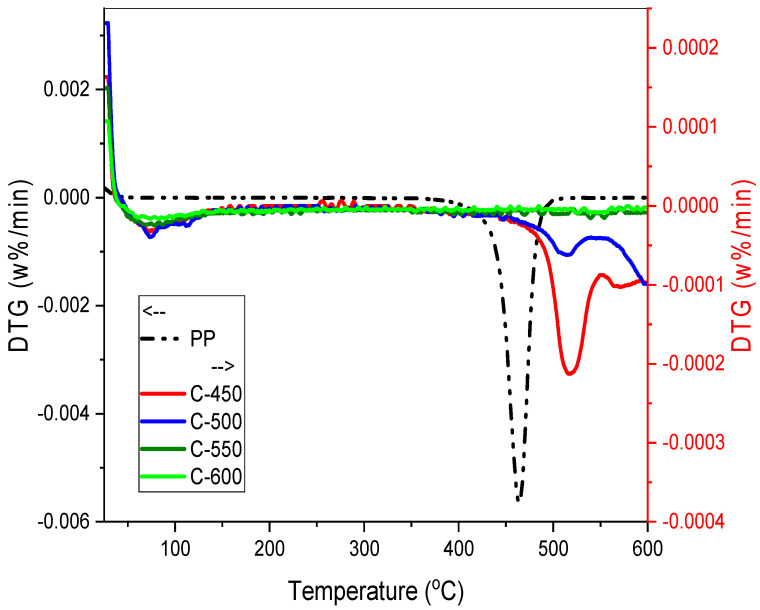
DTG curves for C-450, C-500, C-550 and C-600.

TGA is a common technique for determining weight loss in relation to time or temperature. Thermal stability of PP-IG samples and chars was determined via the TG curves (Figure 5 and Figure 6), under a non-isothermal condition with a heating rate of 10 °C/min in the range of temperature 25 to 600 °C. The degradation of PP-IG samples and PP-IG derived chars are different from each other.

A single degdaration phase can be observed in raw PP-IG samples. As each PP polymer chain, carbon atom consisting of the polymer branching is tertiary carbon, PP-IG deterioration was initiated at a lower temperature of 350 °C, as illustrated in Figure 5 (refer to the dash-dotted black line). This phenomenon was supported by the results of thermal properties of PP analysed by Das et al. [51]. Branching itself weakened the polymer chain, consequently making it more susceptible to degradation compared to a linear polymer of LDPE and HDPE. At temperatures ranging from 350 to 550 °C, polypropylene chain branches and double-bonded backbones decomposed with a weight loss of 97%. In order to compare the thermal degradation of individual plastics, detailed TG analysis and kinetic study of PP [52], LDPE and HDPE [53,54], and PS were studied [55]. The residue left after 550 °C was labelled as carbonaceous char [56].

Practically, the char sample did not heat instantaneously within the first few minutes; thus, errors ascend where the high temperature was too high for significant decomposition to occur. This phenomenon, which was explained by Witkowski et al. [41], took place in the early stage of TGA of chars at the temperature range of 25 to 50 °C, as shown in Figure 5 and Figure 6. The first stage of thermal decomposition of chars, C-450, C-500, C-550, and C-600, at the temperature range of 50 to 150 °C with a weight loss of about 2%, associated with the removal of adsorbed water molecules on the char’s surface. Within the temperature range of 350 to 600 °C, the second phase was interlinked with the condensation reaction of hydrocarbons and the formation of char. This was a vigorous pyrolysis phase marked by a substantial weight loss of roughly 10% and the generation of a large number of light diesel oils and gases. The degradation occurred due to (i) the production of free radicals during the onset of polymer chain breakdown and (ii) the dispersion of volatile degradation products. C-450 and C-500 chars pyrolysed greatly, 9% and 4%, respectively, whereas C-550 and C-600 experienced slight weight loss, 1% and 0.5%, due to their reduced hydrocarbons amount.

#### 3.3.5. Functional Group Analysis

**Figure 7:**
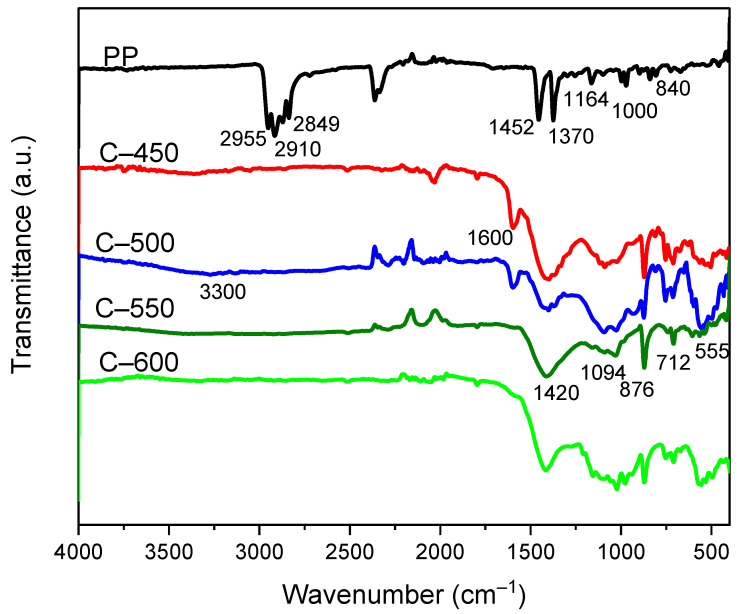
FTIR–ATR spectra of raw PP-IG and chars collected from pyrolysis with different temperatures.

Compositional and functional group analysis of PP-IG and char samples including C-450, C-500, C-550, and C-600, were obtained via FTIR spectroscopy. The signatures of FTIR spectra were observed within the area of the spectrum between 4000 and 400 cm^−1^, as shown in Figure 7, which demonstrated the presence of –CH_3_, –CH_2_ and C–H groups of high carbon content solid products.

The PP-IG samples of ATR-FTIR (Figure 7, PP-IG curves) display absorption peaks in line with the literature data published [57]. Moderate absorption peaks of deformation vibrations of plane CH_2_, in the range of 1440 to 1480 cm^−1^ were emerged, while –CH_3_ groups vibrations were registered in the spectral range of 1440 to 1465 cm^−1^ or 1365 to 1390 cm^−1^, according to the common polypropylene spectrum [58]; whereas within our spectrum, these peaks were recognised at 1452 cm^−1^ and 1370 cm^−1^, respectively. In addition, broad and intense peaks were discovered at 2849 cm^−1^ and 2955 cm^−1,^ where these peaks were attributed to the symmetrical stretching vibration modes of –CH_2_ and asymmetrical stretching vibration modes of –CH_3_, respectively. A distinct and intense band at around 2910 cm^−1^ was associated with the stretching vibration modes of –CH groups. Moreover, the absorption peaks at 840, 1000, and 1170 cm^−1^ represented the characteristic vibrations of terminal unsaturated –CH_2_ groups that were present in isotactic PP as registered in the UCLA Infrared Spectroscopy Table [59]; whereas in our experimental spectrum, the characteristic features were observed at 840, 1000, and 1164 cm^−1^, respectively. Thus, the features of raw PP-IG samples have coincided perfectly with the works from Krylova and Dukstiene [60] about neat PP.

The ATR–FTIR spectrum of PP-IG derived char samples, C-450, C-500, C-550, and C-600, yielded at different pyrolysis temperatures, showed constancy in their functionality and characteristics, as shown in Figure 7. Broad absorption bands with weak intensity at range 3400 to 3300 cm^−1^ corresponded to the hydroxyl–OH groups stretching and bending attributed to the physisorption of moisture adsorbed on the surface of C-450, C-500, and C-550, while being almost flat in the spectrum of C-600 [61]. The presence of C=C stretching vibrations due to aromatic rings was demonstrated in the spectral ranges of 1400 to 1500 cm^−1^ or 1585 to 1600 cm^−1^, and a high absorption peak of stretching vibrations from C=C bonds from alkenes was observed in the spectral range of 1600 to 1680 cm^−1^ [62]; whereas it was recorded in the char spectrum that the absorption peaks were discovered at 1420 cm^−1^ and 1600 cm^−1^, respectively. The intense absorption peak at 1600 cm^−1^ was discovered in spectra of C-450 and C-500 only.

Practical identical elucidations have been discovered in the literature [37,63]. Next, a broad 1094 cm^−1^ absorption band was identical to the hydrocarbon groups, which are mostly comprised of monosubstituted benzene. On the other hand, the peak at 876 cm^−1^ characterised the out-of-plane deformation formed by aromatic C–H atoms [64]. Thus, this coincided with previous works of PP char by Sogancioglu et al. [37]. The author mentioned that the distinct peaks at spectra region between 800 to 900 cm^−1^, 700 to 800 cm^−1^, and 500 to 600 cm^−1^ represented *p*-disubstituted benzene aromatic C–H and alkene groups. From our continuous spectrum, the peaks were observed at 876 cm^−1^, 712 cm^−1^, and 555 cm^−1^, respectively. Conclusively, from the C-550 and C-600 spectra, aliphatic bands and alkene bands were reduced greatly, while aromatic C=C and C–H groups were attained. This was due to the improvement of aromatic structures of chars as the pyrolytic temperature increased. In placing more emphasis, Xiao et al. [65] already explained that the char layers were composed of multi-aromatic carbon. In addition, the presence of functional groups such as carboxyl groups could be used to react with the functional groups of certain polymers, compatibilisers, or binders such as starch to improve interaction with the polymer matrix [66,67].

#### 3.3.6. Raman Spectroscopy

**Figure 8:**
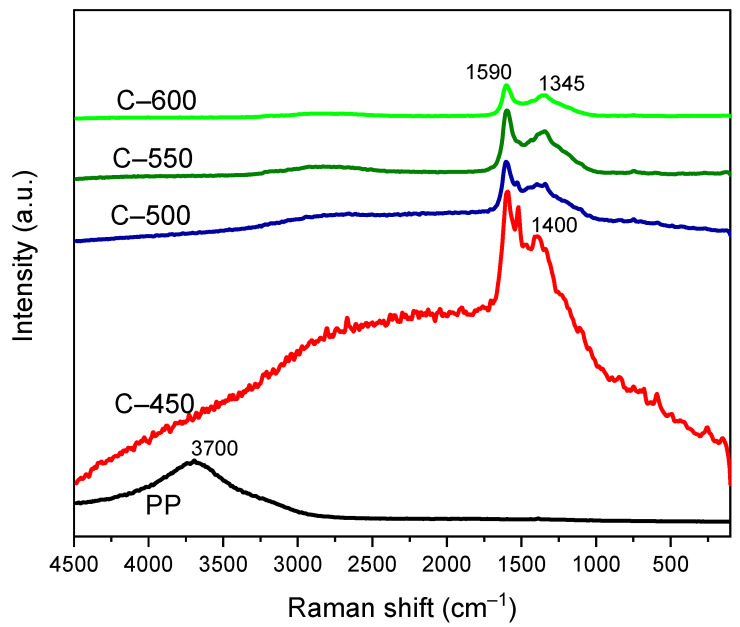
Raman spectra of raw PP-IG and char samples were obtained at different operating temperatures.

Raman spectroscopy and FTIR spectroscopy proved the vibrational spectrum by inelastic scattering and absorption, respectively, for polymer chains conformation [68]. In many cases, these two approaches complement each other. The Raman spectra of neat PP-IG samples was recorded as reference spectra for further study of PP-IG derived char with different elevated pyrolysis temperature, as shown in Figure 8. For pure PP-IG, an intense and broad peak was discovered at 3700 cm^−1^. A similar result had been observed by Bhattacharyya et al. [69] and Ahmad et al. [70]. According to the registered bands in the vibrational spectrum of PP [68], the peak that arose within the Raman frequency range of 2900 to 3700 cm^−1^ was ascribed to the vibrations due to asymmetrical –CH, –CH_2_, and –CH_3_ functional groups. Thus, this result supported the discoveries of the methane, methyl, and methylene groups within the structure of the raw PP-IG samples (refer to Figure 8).

Raman spectroscopy and XRD are two fundamental approaches for the characterisation of an intumescent carbonaceous material [71]. Within the spectra of char samples, there were two distinct peaks discovered of each spectrum at 1590 and 1400 cm^−1^. However, a slight blue-shift took place for C-550 and C-600 char samples, 1345 cm^−1^. Both peaks represented the characteristic of pre-graphitic structures, where according to Tamor and Vassell [72], the first peak was associated with the E vibrational mode, while the second was for structural defects. A similar trend was also observed by Zhou et al. [71] of Raman curves of outer char of PP-based composite. In addition, in terms of intensities, as shown in Figure 8, both peaks showed decreased ‘peaks’ intensity as the process temperature increased. At 1590 cm^−1^, C-450, C-500, C-550, and C-600 recorded a declining trend of intensity peaks with 8609.81, 2503.27, 2265.14, and 1469.06 au, respectively; whereas at the wavelength of 1400 cm^−1^, char sample C-450 and C-500 registered an intensity of peaks of 7750.42 and 2105.12 au, and at 1345 cm^−1^, the char samples C-550 and C-600 logged lower intensity peaks, 1877.66 and 1295.97 au, respectively, due to the deteriorating structural organisation level of the carbon [73].

## 4. Conclusions and Future Outlooks

This study investigated the slow pyrolysis of PP-IG plastic waste using a batch reactor. The thermal decomposition of the pyrolysis model was established with a slow heating rate, long residence time, and lower pyrolytic temperature. It was used to enhance the char yields as the experimental results. The main conclusions of this study are:

(i) The low–temperature pyrolysis of polypropylene-based COVID-19 isolation gown waste (PP-IG) resulted in improved char yields (2.27 wt.%), which mostly comprises aliphatic and carbonaceous alkene structures.
(ii) On the other hand, increasing the temperature decreased the yield of char products but produced char with higher surface area value and enhanced pore volumes, ~24 m^2^g^−1^ and ~0.08 cm^3^g^−1^, respectively.
(iii) The char obtained at higher temperatures (C-550 and C-600) comprised more aromatics C=C and carbonaceous C–H structures due to volatilisation and carbonisation.
(iv) The low temperature (450 °C) and long residence time (5 h) degradation process supported the reaction from the polymer scission and led to a lighter hydrocarbon with reduced microporosity due to lower carbon structure deterioration.
(v) In order to generate unique char products from PP-IG waste, data collected during this study are valuable for the use of pyrolysis. At the same time, further studies are needed in order to optimise the pyrolysis parameters, design, and catalyst support for better pyrolysis outputs.
(vi) In addition, the isothermal process used less energy and not an isothermal process, making the present work more reliable and practical in terms of transforming COVID-19 related plastic waste into energy.

Char yielded under low-temperature pyrolysis from PP-IG waste can fulfill the demand for alternate fuel such as barbeque application and also utilization of plastic waste, especially during the COVID-19 pandemic. Even after the end of the pandemic, this study is still practical to be used to treat other PP-based plastic waste, that of PP being one of the highest produced plastics globally.

In terms of waste treatment, the practice of slow pyrolysis of PP-based plastic waste has shown numerous positive impacts. This is due to the fact that this study has successfully demonstrated how easy the process design is, with pyrolysis and heating rate optimisations, to convert PP plastic waste originated from used PPEs into multi–functional carbonaceous products. Furthermore, this research may also add to the effort to uncover the potential of plastic-derived char as solid precursors in developing green char fuel briquettes products and graphene/activated carbon production, as there are such abundant plastic waste resources were underutilized.

However, to be employed for domestic use, health considerations must be taken into account. Thus, toxicology characteristics and further effects from its applications towards the environment and human health must be studied extensively. These considerations will provide a better practicality point for this study for applications even after the end of the pandemic. The utilization of hazardous PP plastic waste will contribute to plastic waste reduction and better environmental health.

## Figures and Tables

**Table 1:** Elemental composition of PP plastic.

Element	C	H	N	O	S
**Amount (wt.%)**	87.85	10.58	0.23	0.48	0.86

**Table 2:** Proximate analysis for raw PP-IG samples.

Sample	Moisture (wt.%)	Volatile Matter (wt.%)	Ash Content (wt.%)	Fixed Carbon (wt.%)
PP-IG	0	95.75	0.38	3.87

**Table 3:** Char yields (wt.%).

Samples	C-450	C-500	C-550	C-600
**Temperature (°C)**	450	500	550	600
**Char yields (wt.%)**	2.27 ± 0.45 ^a^	2.12 ± 0.25 ^a,b^	2.06 ± 0.08 ^a,b^	1.97 ± 0.04 ^b^

^a,b^ values with different letters in the same column are significantly different (*p* < 0.05).

**Table 4:** Moisture and volatile matter contents for chars.

Samples	C-450	C-500	C-550	C-600
**Moisture content (** **wt.%)**	0.53	0.67	0.50	0.39
**Volatile matter (wt.%)**	9.47	5.22	2.04	1.39

**Table 5:** Contents of elements from EDX.

Sample	Element Line (wt.%)					
	C K	O K	Ca K	P K	Al K	K K
PP-IG	95.67	4.00	0.32	-	-	-
C-450	27.23	38.87	22.24	10.55	0.66	0.46
C-500	16.09	44.36	27.10	11.53	0.47	0.44
C-550	17.45	46.32	30.53	4.03	0.60	0.57
C-600	14.11	40.55	25.67	18.77	0.43	0.47

**Table 6:** Specific surface area and characteristics of the porous structure of raw PP-IG and yielded chars.

Sample	SBET (m^2^ g^−1^)	Dp (nm)	Vp (cm^3^ g^−1^)
PP-IG	4.98	28.11	0.0350
C-450	17.65	10.86	0.0479
C-500	12.81	20.30	0.0650
C-550	25.25	11.75	0.0742
C-600	22.91	14.03	0.0804

## Data Availability

Not applicable.
